# Communicating with patients requiring anti-VEGF intravitreal injections and their families during the COVID-19 pandemic: an update

**DOI:** 10.1007/s00417-020-05042-7

**Published:** 2021-01-07

**Authors:** Jean-François Korobelnik, Anat Loewenstein, on behalf of the Vision Academy

**Affiliations:** 1 Service d’Ophtalmologie, CHU Bordeaux, Bordeaux, France; 2 Inserm, Bordeaux Population Health Research Center, team LEHA, Université de Bordeaux, UMR 1219, F-33000 Bordeaux, France; 3 Division of Ophthalmology, Tel Aviv Medical Center, Sackler Faculty of Medicine, Tel Aviv University, Tel Aviv, Israel

Dear Editor,

In Spring 2020, with our colleagues at The Vision Academy [1], we identified the need for clinician guidance on how best to communicate with patients and caregivers to ensure crucial eye care appointments and services were continued during the current COVID-19 pandemic. At that time, we noted, in particular, heightened anxiety levels among patients, receiving intravitreal injections of anti-vascular endothelial growth factor (VEGF) for neovascular age-related macular degeneration (nAMD), who need to attend regular ophthalmology appointments to maintain their vision. We therefore published guidance for managing such patients during the acute phase of the COVID-19 pandemic [2] and supported this with a patient communication template. The communication could be sent via e-mail, post, or text messages ahead of appointments to reassure patients and caregivers that their safety and eye health remained a priority [3].

Since then, the COVID-19 pandemic has escalated: clearly, it is not going to disappear quickly, and we need to make the necessary adjustments to establish a routine of safely treating patients while the pandemic is ongoing. Evidence has emerged that people with moderate/severe visual impact are significantly more likely to experience worsened mental health because of COVID-19-related restrictions than those with mild/no visual impairment [4]. Additionally, a UK study found that approximately 50% of patients with nAMD failed to attend a scheduled hospital or clinic appointment [5]. Of those non-attendees, 85% said they were fearful of contracting COVID-19, and over 70% would have attended if they had been given clear advance information of adequate infection control measures. These findings are understandable given patients with nAMD are at increased risk of COVID-19-related complications, hospitalization, and mortality because of increased age with comorbid conditions. They also reinforce the need for patient information tools.

We now provide greater detail about the implementation of patient guidance according to the local epidemic situation, based on three (risk) tiers, until such time that long-term solutions (e.g., effective vaccine) are available [6]:

1. The effective reproduction number, *R*_*t*_, is < 1, but herd immunity through mass vaccination has not yet been achieved
2. *R*_*t*_ is ≈ 1, and local COVID-19-positive clusters result in a relatively high risk of contracting COVID-19, although local hospital resources are not critically overwhelmed
3. *R*_*t*_ is > 1, and hospital resources are under current or imminent risk of significant pressure, with higher number of local or national restrictions likely

For each scenario, we provide guidance on prioritizing patients according to clinical need, while minimizing risk for patients and healthcare professionals.

**Fig. 1:**
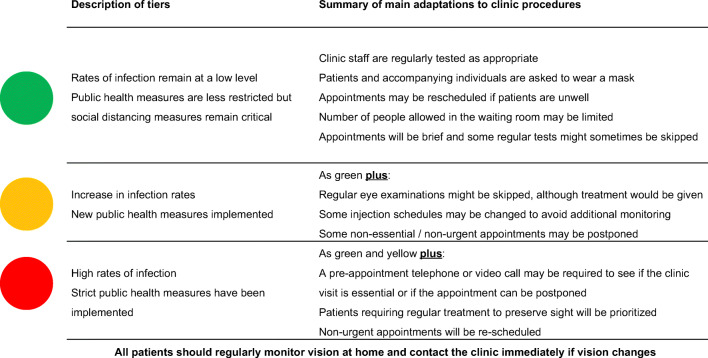
Description of main clinic procedural adaptations during the COVID-19 pandemic according to local epidemic situation, summarized from the patient infographic

The Vision Academy has also endorsed a new ‘Dear Patient’ letter and two accompanying infographics with the goals of clear communication to minimize patient/caregiver anxiety and maximize clinic attendance. Three color-coded tiers (green, yellow or red) indicate simply the level of risk and, most importantly, the precautions that are in place to ensure the safety of patients and their caregivers (Fig. 1). It is very important that appointments are not missed especially during treatment phases.

Educational material in the form of infographic patient factsheets highlights characteristics for each of the three risk scenarios (and include an Amsler Grid) and provide patients with information on maintaining and improving their mental wellbeing alongside their eye health. When patients are unable to attend the clinic, they must re-schedule the appointment as quickly as possible and monitor their vision in the meantime. Should any change in their vision occur, patients are asked to re-contact the clinic.

We encourage the ophthalmic community to download these patient materials from https://www.visionacademy.org/vision-academy-community/COVID-19-materials and adapt them for local use. Regionally, campaigns to call patients and encourage them not to miss examinations and treatments should be considered.

Prof. Jean-François Korobelnik

Prof. Anat Loewenstein
